# A simple metric for a complex outcome: proposing a sustainment index for health indicators

**DOI:** 10.1186/s12913-018-3340-2

**Published:** 2018-07-11

**Authors:** Eric Sarriot, Reeti Desai Hobson

**Affiliations:** 1 Save the Children, Department of Global Health, Washington DC, USA; 2 ICF, International Health and Development Department, 530 Gaither Road, Suite 500, Rockville, MD 20850 USA

**Keywords:** Sustainment index, Sustainability, Evaluation research, Program evaluation, Post-project evaluation, Health systems strengthening, Wicked problems, Complex adaptive systems

## Abstract

**Background:**

Sustainability is, at least in principle, an important criterion for evaluating global health and development programs. The absence of shared metrics for success or achievements in sustainability is however critically lacking. We propose a simple metric, free of causal inference, which can be used to test different empirical models for the sustainment of health outcomes.

**Methods:**

We follow the suggestion of Chambers and use “sustainment” to refer to the verifiable and measured extent to which a health indicator has evolved over time. The sustainment index of a health indicator (Y) advanced by a program is based on a simple-to-calculate approximation of the derivative of Y over time (T0: baseline, T1: endline, and T2: post-project), based on the ratio of the slope of Y_T1-T2_ over Y_T0-T1_. SI(Y) = 1+ (Y_T1-T2_ / Y_T0-T1_).

**Results:**

This construct provides three clear benchmarks: SI = 0, when the health indicator returns to baseline value post-project (Y_T2_ = Y_T0_); SI = 1, when the endline-post-project trend is a plateau; and SI = 2, when the progress slope during program is uninterrupted post-program. We find strong correlation (r^2^ = 0.922) between the SI and independent practitioners’ rating of indicator trends. The SI shows different levels of achieved sustainment for a range of indicators in a published ex-post sustainability study. And we find that the SI can be computed for large national datasets for two types of indicators.

**Conclusions:**

The Sustainment Index has limitations and conditions of applicability, but it can be applied to different datasets and studies to provide a reliable dependent measure of the level of sustainment of health outcomes from one period of time to the next. The Index will need additional testing, and future evaluation-research work will need to consider index performance under different situations. The Sustainment Index has the potential to provide a standard metric to build evidence through more systematic research on sustainment and sustainability.

## Background

Sustainability is, in theory, an important criterion for evaluating development and health programs. In this context, sustainability refers to what happens to communities and beneficiaries after external funding ends; institutionalization of practices within local social or institutional systems; or maintenance of achieved outcomes [1–5]. A lot of efforts have focused on the conceptualization of sustainability [6–8] and surveys of program implementers to identify key factors of sustainability [9], with empirical evidence in the form of post-project sustainability studies very slowly emerging [9–12].

Authors bemoan the slow empirical progress in sustainability evaluation and evaluation-research in global health, and sometimes place the blame on poor conceptualization [6–8]. Given the challenges with rigorous evaluation of how much outcomes have been sustained [10, 13, 14], rather than perception of, or opinions about sustainability, this leads us to an excess of conceptual models and a dearth of empirical studies. While we confess contributing to this conceptual inflation in the past, this paper is concept-neutral and proposes a simple new metric, which we hope other researchers will use in order to stimulate more empiricism. First, we seek to clarify two things with our language.

Sustainability is unfortunately often used as a self-contained word, representing aspirations, values, or a vision. In global health, the question “what about sustainability?” can introduce almost any discussion: cost, cost-recovery, governmental institutionalization, country ownership, partnerships, accountability, organizational capacity, policies, the “enabling environment,” supply and demand, unspoken assumptions about private versus governmental versus civil society responsibilities, and the latest disappointments of the discussants in the room. Our focus will be on the measured sustainment (see below for this term) of health outcome indicators, or acceptable proxies, such as immunization coverage. While we believe that sustainability is a non-linear, highly contextual, and process oriented [15], we do not address these questions in this paper. When we focus on health outcomes, the question becomes how much was a benefit sustained, and how did this happen?

Secondly, *sustain-ability* refers to the ability to make something last into the future. Confusingly, the same term is also used to speak about how much a benefit has actually been sustained over time. Chambers [6] proposes to use the word “sustainment” to speak of that which can be observed looking back in time. We chose to follow Chambers convenient suggestion. Accordingly and for the rest of this paper:

- *Prospectively*--Sustainability refers to the ability, or potential of an entity (local system, health system, organization, etc.) to maintain a function or public good.
- *Retrospectively*--Sustainment refers to the verifiable extent to which a public good has measurably evolved over a time.

## Method

### Identifying a dependent variable

We now present the construction of the sustainment index for health indicators, as the dependent variable against which different models can ultimately be tested, and three analytical steps, which we have taken to validate its construct. Operationally, we want to measure how a health indicator (Y) trend from time T0 to T1 continued from time T1 to T2. The sustainment index is a simple-to-calculate approximation of the derivative of Y over time (T0: baseline, T1: endline, and T2: post-project), based on the ratio of the slope of Y_T1-T2_ over Y_T0-T1_. The sustainment index is a quantification of a trend change over two period for a health indicator—it carries no assumptions about human agency, program contribution, attribution, secular trend, or other (see Discussion).

### Part 1 - development of the sustainment index

**Fig. 1:**
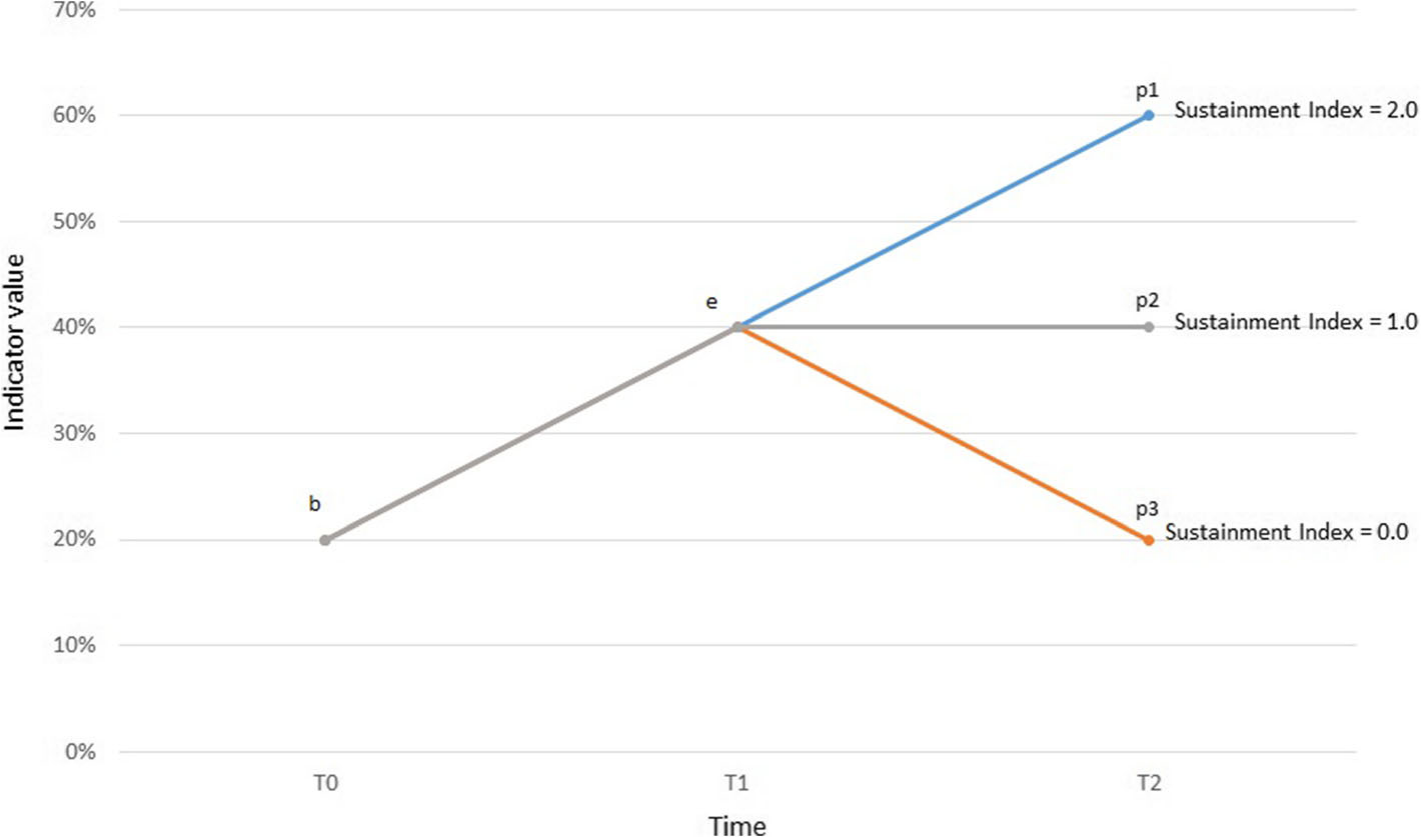
Health Indicator Progress through Three Points in Time

We start with a draft index, which we then modify for boundary conditions. Figure 1 presents the evolution of the value of Y, independent of causal interferences, across three points in time:

- *b* = Y_T0_: baseline value of Y;
- *e* = Y_T1_: endline value of Y; and
- *p* = Y_T2_: post-project value of Y.

We treat the evolution of Y across the two periods as a linear trend, and calculate the ratio of the slopes (Y_T1-T2_ / Y_T0-T1_). This assumes that the endline estimate was greater than the baseline in a statistically significant manner (if the original indicator trend was negative, the issue would be how to take corrective measures, not how to sustain). We add 1 to calibrate the sustainment index around three benchmarks for the value of *p* (Fig. 1):

- *p1*: Sustainment Index = 2.0. Flawless continuation of progress from T0 to T1 until T2.
- *p2*: Sustainment Index = 1.0. Plateau reached after T1.
- *p3*: Sustainment Index = 0. Return to baseline conditions, no sustainment.

This leads us to a first draft of the index:

1. Sustainment Index Condition of Applicability: *e* > *b*:
  $$ \mathrm{Index}\ \mathrm{Draft}=1+\frac{\left(p-e\right)/\Delta 2}{\left(e-b\right)/\Delta 1} $$
  With ∆1 = T1-T0 and ∆2 = T2-T1

Values of the Sustainment Index above 2.0 and below 0.0 are possible, and would correspond respectively to an acceleration of progress beyond mere sustainment, or a reversal of progress beneath baseline conditions.

Two “boundary conditions” of the indicators under study could affect the value of the Sustainment Index and need to be addressed.

#### Natural boundary A

As an illustration, if *b* = 70% and *e* = 90%, then a sustainment index value of 2.0 would require *p* = 110%, which is impossible. In this case, we recalibrate our measure so that the maximum plausible value of *p*, *p*max = 100% (at T2) gives us the maximum plausible value of Sustainment Index (2.0). This natural boundary A is encountered when two conditions are present at the same time:

[C2] - First condition for natural boundary A: $\left(e-b\right)>\left( pmax-e\right)\ast \left(\frac{\Delta 1}{\Delta 2}\right)$, and
[C3] - Second condition: Sustainment Index value is above 1.0 (otherwise, recalibration is not needed): *p* > *e*

**Fig. 2:**
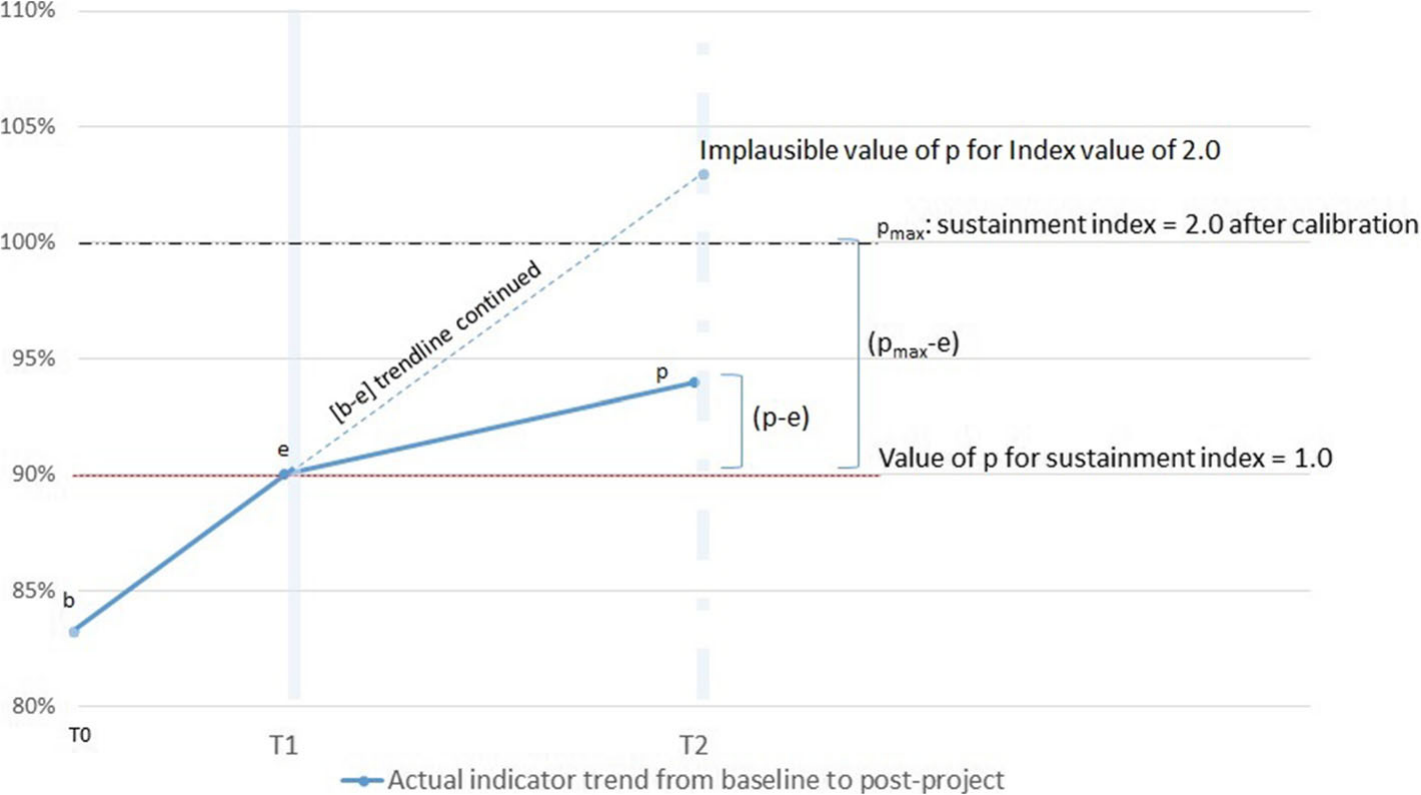
Need for Calibration of Sustainment Index at High Values that Cross *b-e-p* Trend Line

If C2 and C3 are respected then the sustainability index term $\frac{\left(p-e\right)}{\left(e-b\right)}$ needs to be replaced by $\left(\frac{p-e}{pmax-e}\right)$. Figure 2 illustrates this visually.

#### Natural boundary B

For some indicators, *p*max cannot feasibly be 100%. This can be seen in the example of the Contraceptive Prevalence Rate (CPR), for which a maximum of 65% can be set for all practical purposes. The same should apply to other indicators.

This provides us with the final conditional equations for the Sustainment Index, whereby accounting for conditions C1 to C3 addresses the two possible boundary conditions.

Conditional Equations for the Sustainment Index:

1. *e* > *b*
2. $$ \left(e-b\right)>\left( pmax-e\right)\ast \left(\frac{\Delta  1}{\Delta  2}\right) $$
3. p > e

- If C1 = false, the Sustainment Index is not applicable
- If C2 = true and C3 = true$\mathrm{Sustainment}\ \mathrm{Index}=1+\left(\frac{p-e}{pmax-e}\right)$
- If C2 = false or C3 = false$\mathrm{Sustainment}\ \mathrm{Index}=1+\left(\frac{p-e/\Delta  2}{e-b/\Delta  1}\right)$

### Part 2 – Testing the sustainment index

We then tested the validity of the Sustainment Index metric through:

- A face validity validation exercise;
- Application to a real post-project dataset; and
- Application to Demographic and Health Surveys (DHS).

### Correlation of the sustainment index with an independent practitioners’ panel rating

We opportunistically selected a panel of seventeen health professionals, each with a Master’s in Public Health, MD or PhD degree, and with five to 30 years of global health work with various bilateral and government agencies, non-governmental organizations (NGOs), and in-country partners, to provide expert ratings and correlate those with computed values of the Sustainment Index.

**Fig. 3:**
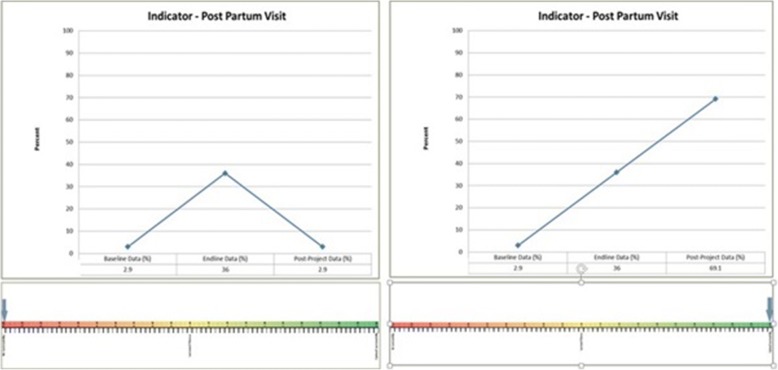
Graduated Visual Scale - No Sustainability (SI = 0, return to baseline) and Trend in Improvement Unchanged (SI = 2)>

We divided the experts into groups of two or three and assigned to each group a set of baseline and endline maternal, newborn and child health indicators, and hypothetical post-project indicator data from a hypothetical district. Panel members had no information on why an indicator had improved in the first place, or on why it progressed in a certain direction during the second phase of measurement. Each group only had to answer how much improvement of an indicator from T0 to T1 had been sustained from T1 to T2, using a graduated visual scale ranging from 0 (return to baseline) to 2 (unchanged trend). We provided the same two examples to each group to calibrate overall responses (Fig. 3). Experts provided ratings on a total of 61 indicator sets, comprised of baseline, endline, and hypothetical post project values. We used the Pearson correlation coefficient to compare the expert group rating with our measure.

### Application of the sustainment index metric to a published pre- and post-project dataset in Bangladesh

We then used actual and published baseline, endline, and five-year post-project data on an urban health project carried out in two Bangladeshi municipal health departments (Saidpur and Parbatipur) [16]. We applied the Sustainment Index construct to 13 reported health indicators, and observed the distribution of the Sustainment Index values.

### Application of the sustainment index metric to demographic and health survey datasets

The DHS [17] provides internationally standardized nationally-representative population-based datasets. We applied the Sustainment Index to two maternal and child health outcome indicators:

1. Contraceptive Prevalence Rate (CPR): percent of currently married women using any family planning method; and
2. Percent of children aged 12–23 months who have received all basic vaccines by two years of age (Full Vaccination).

We used data from 22 countries in Africa with at least three waves of DHS surveys conducted between 1994 and 2014 (Table 2) [17]. The three waves of DHS surveys were treated as baseline (T0 – earliest survey), endline (T1), and post-project (T2 – latest survey) values for each indicator. We kept the convention of referring to indicator values as *b*, *e*, and *p*, even though the terms “baseline”, “endline”, and “post-project” do not apply to these national longitudinal data. Our goal was simply to describe the behavior of the metric. We set *p*max at 100% for immunization. For CPR we used *p*max = 65% based on the highest observed values in African countries [18].

## Results

### Correlation of the sustainment index with an independent practitioners’ panel rating

**Fig. 4:**
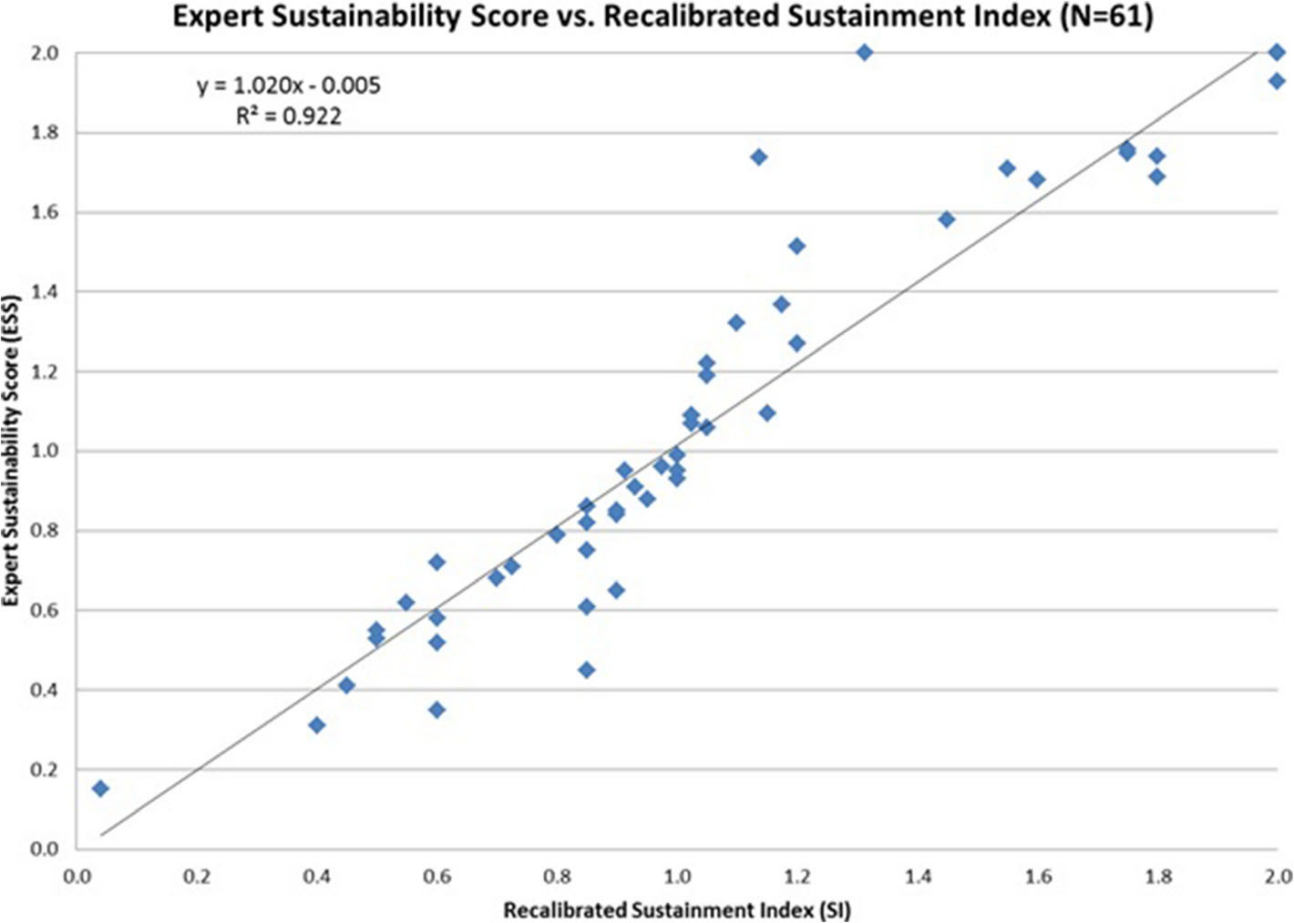
Expert Sustainment Score versus Sustainment Index

The Pearson correlation coefficient (r^2^) between the expert score and our first draft measure was 0.621. After re-calibrating our measure for boundary conditions C2 and C3 (final Sustainment Index) we obtained an r^2^ value of 0.922 (Fig. 4), which indicated strong correlation.

### Application of the sustainment index metric to a published pre- and post-project dataset in Bangladesh

Eight indicators (Table 1), of the eleven child health and maternal and neonatal health indicators analyzed, had a Sustainment Index within the range of 1.0 and 1.6, matching the expected profile of indicators plateauing during the post-project period described by Sarriot [9]. Two indicators had a Sustainment Index of 0.0 and 0.5, effectively corresponding to return to close-to-baseline conditions.

**Table 1:** Application of the Sustainment Index to an Actual Post-Project Dataset in Bangladesh [9]

	Indicator	1999	2004	2009	Sustainment Index (2009)
Child Health	Complete Immunization	44%	91%	91%	1.00
	Vitamin A Supplementation	37%	78%	79%	1.02
	Exclusive breastfeeding	55%	72%	73%	1.06
	Complementary feeding of children 6 to 11 months	*46%*	64%	65%	1.06
	Additional feeding and fluids for the sick child	25%	44%	25%	0.00
	Proper child acute respiratory infection (ARI) identification and referral	24%	34%	52%	2.80
Maternal & Neonatal Health	At least one prenatal consultation during last pregnancy	59%	89%	95%	1.20
	At least one tetanus toxoid (TT) dose during last pregnancy	46%	89%	70%	0.56
	Delivery by skilled attendant	31%	50%	59%	1.47
	Delivery in health care facility	25%	45%	57%	1.60
	Immediate breastfeeding	26%	57%	64%	1.23

Qualitative data in the source publication address the question of attribution, but the Sustainment Index helps us identify a distribution of indicators, with different degrees of sustainment. In effect, our index shows potential for helping us ask why different indicators behaved differently in a post-project period, and helps us discriminate between relatively higher or lower levels of sustainment.

Proper identification and treatment of Acute Respiratory Infection (ARI) is an outlier with a Sustainment Index of 2.8, which corresponds to a phase of accelerated improvement where gains sustained continue to increase in the post-project period. The initial publication identified this indicator as based on a relatively small sub-sample of children, thus putting into question whether this measured acceleration is realistic or a measurement artifact of the original indicator. The reliability of the underlying indicators is central to the reliability of the Sustainment Index.

### Application of the sustainment index metric to demographic and health survey datasets

Table 2 presents our findings for all countries for both indicators under study.

**Table 2:** Sustainment Index for 22 countries, 2 indicators and 2 time periods [17]

Country	Indicator	T0 (Base %)	T1 (End %)	T2 (Post %)	Sustainment Index
Benin	CPR	7.2	6.1	7.9	C1 not met
	Immunization	59	47.1	47.6	C1 not met
Burkina Faso	CPR	4.8	8.8	15	1.89
	Immunization	29.3	43.9	81.3	2.46
Cameroon	CPR	7.1	12.5	14.4	1.30
	Immunization	35.8	48.2	53.2	1.35
Cote d’Ivoire	CPR	4.3	7.3	12.5	1.67
	Immunization	37.4	50.7	50.5	0.99
Ethiopia	CPR	6.3	13.9	27.3	2.47
	Immunization	14.3	20.4	24.3	1.53
Ghana	CPR	13.3	18.7	16.6	0.61
	Immunization	62	69.4	79	2.30
Guinea	CPR	4.2	5.7	4.6	0.37
	Immunization	32.2	37.2	36.5	0.88
Kenya	CPR	31.5	31.5	39.4	C1 not met
	Immunization	59.5	51.8	68.3	C1 not met
Lesotho	CPR	35.2	45.6		T2 not available
	Immunization	67.8	61.7		C1 not met
Madagascar	CPR	9.7	18.3	29.2	2.77
	Immunization	36.2	52.9	61.6	1.73
Malawi	CPR	26.1	28.1	42.2	5.70
	Immunization	70.1	64.4	80.9	C1 not met
Mali	CPR	5.7	6.9	9.9	2.79
	Immunization	28.7	48.2	38.9	0.66
Mozambique	CPR	5.1	20.8	11.3	0.55
	Immunization	47.3	63.3	64.1	1.04
Namibia	CPR	42.6	53.4	55.3	1.21
	Immunization	64.8	68.7	68.4	0.91
Niger	CPR	4.6	5	12.2	25.00
	Immunization	18.4	29	52	3.89
Nigeria	CPR	8.2	9.7	9.8	1.07
	Immunization	12.9	22.7	25.3	1.27
Rwanda	CPR	10.3	27.4	45.1	2.55
	Immunization	75.2	80.4	90.1	3.80
Senegal	CPR	12.1	16.1	20.3	3.10
	Immunization	62.8	70.2	73.7	1.95
Tanzania	CPR	16.9	20	27.4	3.86
	Immunization	68.3	71.1	75.2	2.76
Uganda	CPR	18.2	17.9	26	C1 not met
	Immunization	36.7	46.2	51.6	1.57
Zambia	CPR	25.3	32.7	44.8	2.17
	Immunization	70	67.6	68.3	C1 not met
Zimbabwe	CPR	92	93	89	−4.60
	Immunization	64	52.6	64.5	C1 not met

Three countries did not meet the conditions of applicability C1 for computing a Sustainment Index for either indicator (Benin, Kenya, Lesotho); three more did not meet condition C1 for immunization (Uganda, Zambia, Zimbabwe), and Malawi did not meet the condition for CPR. Our recalibration for boundary conditions was not needed for this dataset.

Further observations are made by looking at each indicator closely.

**Fig. 5:**
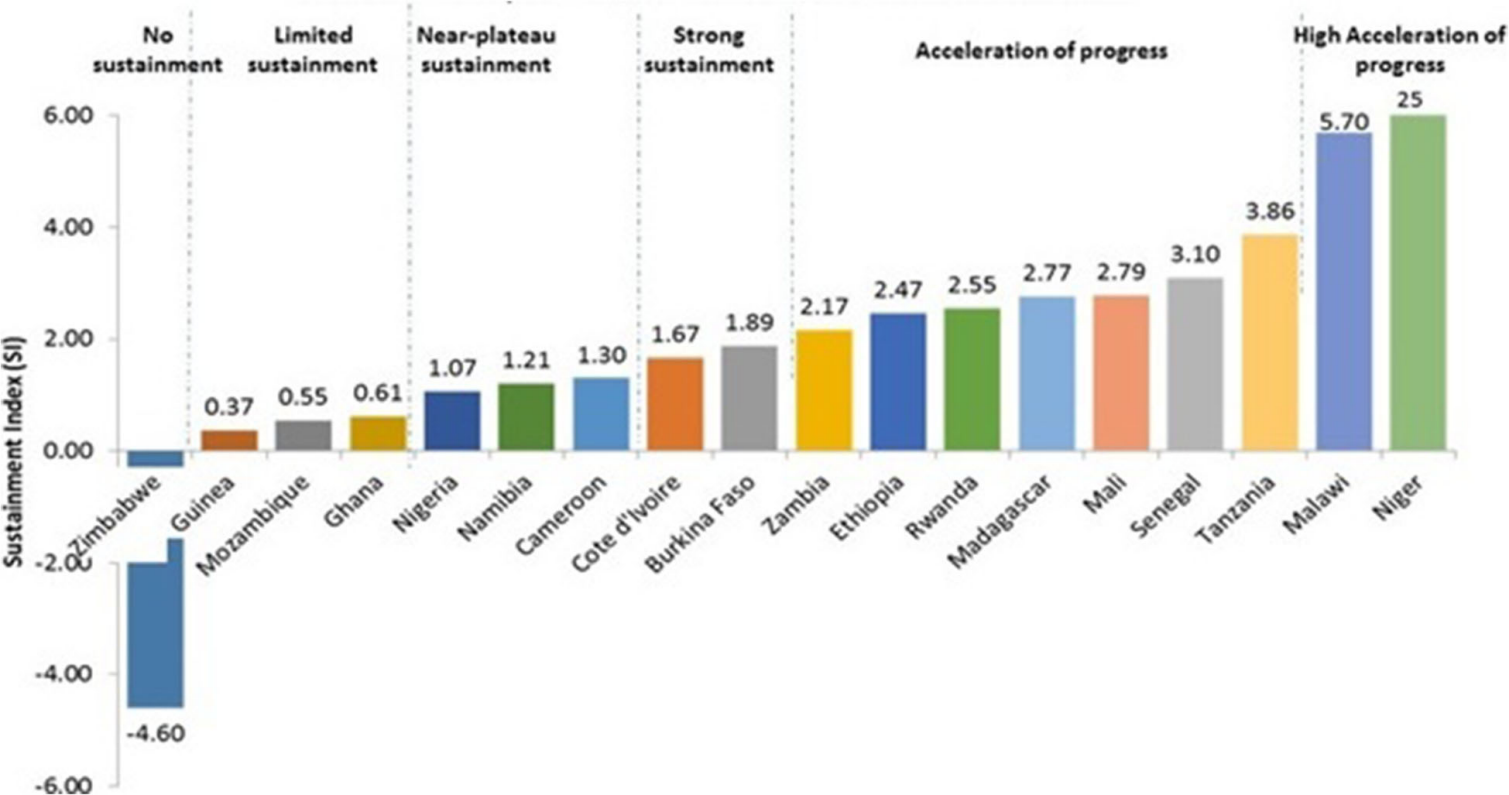
Sustainment Index for Contraceptive Prevalence Rate among Women in 18 Countries [17]>

The Sustainment Index values for CPR in 18 countries ranged from − 4.60 for Zimbabwe to 25.0 for Niger (Fig. 5). One country (Zimbabwe) indicated absolutely no sustainment of CPR and actually showed a reversal to below T0 values, three countries showed limited sustainment (Guinea, Mozambique, Ghana), three more had near-plateau sustainment (Zimbabwe, Nigeria, Namibia), three showed stronger sustainment (Cameroon, Cote d’Ivoire, Burkina Faso), and the remaining eight showed accelerated progress (Zambia, Ethiopia, Rwanda, Madagascar, Mali, Senegal, Tanzania, Malawi, Niger).

**Fig. 6:**
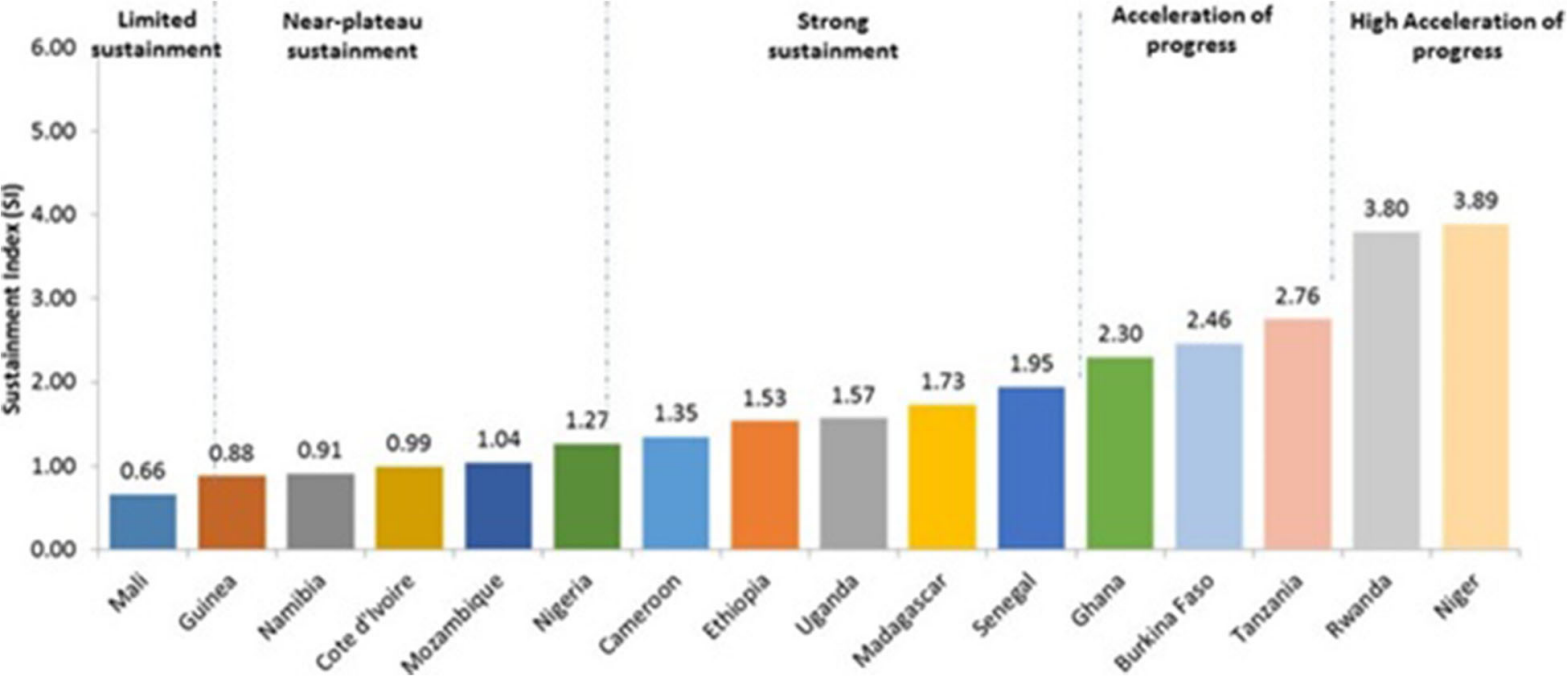
Sustainment Index for Full Vaccination Coverage among Children under Two in 16 Countries

Sixteen countries out of 22 had results that allowed for computation of a Sustainment Index for full vaccination coverage. One showed limited sustainment (Mali), five had near-plateau sustainment (Guinea, Namibia, Cote d’Ivoire, Mozambique, Nigeria), five indicated strong sustainment (Cameroon, Ethiopia, Uganda, Madagascar, Senegal) and five showed accelerated progress (Ghana, Burkina, Tanzania, Rwanda, Niger) (Fig. 6).

## Discussion

### Limitations

This paper offers a metric and presents data to support its face, construct, and computation validation, but it clearly faces a number of limitations.

We are able to understand distribution of level of sustainment, and discriminate between different levels of performance, but the index does not address what allowed progress to be sustained at the measured level. Further research will indicate how much the Sustainment Index adds to our toolbox of measures.

Excessive sensitivity of the measure to small variation between *b* and *e* should be mitigated by respecting conditionality [C1], which stipulates that *e* must be greater than *b* in a statistically significant manner.

One must remember that the index is not a measure of absolute good, but relative inflection of a trend between T0 and T1. It is insensitive to the actual values of the indicator. Progress on coverage from 20 to 40% to 60% would yield the same exact Sustainment Index (2.0) as progress from 20 to 25% to 30%. In other words, the sustainment index measures the level of sustainment of a performance (how the slope of progress is sustained), not the performance of the indicator (the inclination of the trend).

It is the relative value of the Sustainment Index within empirical studies that can be informative (see below). The expected most desirable value of the Sustainment Index will vary in a number of conditions:

- Initial conditions: as baselines (*b*) progress toward their optimum value, progress from *b* to *e* slows down. This may affect the Sustainment Index in different ways. Caution should be exercised in comparing achieved sustainment under different initial conditions.
- Natural and secular indicator trend differences: some indicators tend to follow unidirectional changes under strong secular trends, while others are more subject to the vagaries of government commitment, health care financing, development assistance, and innovations, which can affect them upward or downward. The Sustainment Index cannot be interpreted as an absolute value, absent understanding of context, and a theory of change. (We derived no conclusion about causation from Fig. 6, merely observed and described through quantification of shifts in trends.)

### Value of the sustainment index for future research

The application of a Sustainment Index in Bangladesh for a local intervention with clear start and end dates, and with a rigorous evaluation, allowed for a simple interpretation of the Sustainment Index. Application to national datasets would require different types of research questions with in-depth review of multiple programs, funding streams, and externalities. Where documentation has already been made of project achievements on national scale, the Sustainment Index could be suggested to expand learning. While questions of attribution versus contribution fit the definition of a “wicked” problem when it comes to sustainment, comparisons to secular trend lines have been used in the literature and would allow researchers to strengthen the evidence [9, 19].

The Sustainment Index provides a measure, which can discriminate between different dynamics of change of an indicator from one period of time to the next. It allows comparisons of the behavior of different indicators in the same context, as well as the comparison of the behavior of the same indicator in different contexts—something evidently more useful if the researcher knows something about the contexts and factors at play. Its merit is in providing a quantification of an outcome, which appears desirable – continuation of progress under changing conditions – and in allowing comparison within sensible and empirical parameters. A sustainment index of 1.48 is not “good” or “bad”, but under empirical conditions, ceteris paribus, it is better than an index value of 0.98. The purpose of research is to ask ‘why?’ Ultimately, the sustainment index can be at best one more tool in our analytical toolbox, starting with observations, comparisons, and raising more questions.

In the case of post-project (ex-post) evaluation studies, it will be interesting to systematically examine the distribution of the Sustainment Index value across contexts, projects, interventions and indicators. Large datasets are not likely to be available, but case study post-project evaluation could be enriched by reference to a common metric. This should encourage an increase in the number of post-project evaluations so far carried out and better aggregation of their findings [6, 9–12].

National datasets, too, could be studied using adapted questions, enabling researchers to achieve more power given the larger data pool available. The Sustainment Index could serve as the dependent variable of structured mixed-method studies, by improving categorization of level of sustainment achieved across settings and indicators. It could also inform studies of sustainability through a quantitative systems dynamics lens, designed to analyze iterative change. Different Sustainment Index values in national datasets could also be compared to:

- Contextual baselines and changes;
- Major shifts in program investments;
- Changes in balance of sources of health care financing for different health interventions;
- Delayed (lagged) effects of capacity strengthening, health system strengthening efforts, policy and political changes on future sustainment indices of relevant indicators;
- Differences within countries for indicators receiving different types of support;
- Etc.

Studies could also examine how progress is sustained after the end of external investments across different groups of population, based on income, residence, ethnic identification, or gender, in order to identify pro-equity policies and design factors.

Ultimately, quantifying the degree of sustainment of program investments only makes sense if research is sought to guide policy and program investments. This would make greater sense to development assistance partners seeking to support lower and middle income countries, or to countries seeking to support more poorly performing regions or districts. It would have limited value when conditions are close to satisfactory and at equilibrium.

## Conclusions

The Sustainment Index provides a reliable dependent measure of the level of sustainment of health indicators, which we have tested against different datasets. Even with some limitations, this tool may provide a useful resource to more systematically build the body of evidence on improving the sustainment of health outcomes, and ultimately the sustainability of health program designs, national or international. Future research and evaluation-research applications are needed to consider how it will perform under different situations, how it can be improved, and ultimately whether it allows accumulation of sustainment data and evidence for sustainability through systematic analyses.

Different models and research approaches will contribute to advances in the study of sustainability at scale, and resilience, but we hope that this simple metric can help abolish the question “is *it* sustainable?” in favor of an empirically-testable ones, such as “how much was sustained?” and “how?”

## Abbreviations

ARI: Acute Respiratory Infection
CPR: Contraceptive Prevalence Rate
DHS: Demographic and Health Surveys
NGO: Non-Governmental Organization
SI: Sustainment Index
